# A scoping review of simulation-based dental education

**DOI:** 10.15694/mep.2020.000036.1

**Published:** 2020-02-27

**Authors:** Denise Higgins, Melanie Hayes, Jane Taylor, Janet Wallace

**Affiliations:** 1 University of Newcastle; 2 The University of Sydney

**Keywords:** Dental education, simulation, psychomotor skills.

## Abstract

This article was migrated. The article was marked as recommended.

In order to meet the needs of student learning within a competency-based pedagogy, it is necessary to understand the current philosophies and modalities being used in dental and oral health simulation-based education. The aim of this literature review is to identify the existing research relating to the educational structure of dental and oral health simulation activities. The review is presented as a scoping review, formulated and conducted using a modified five stage methodological framework. Despite evidence showing that the healthcare simulation model is ideal for learning and assessments of non-clinical and clinical tasks such as psychomotor skills, there is a paucity of published literature relating to simulation in dental and oral health education. Out of 72 initial articles only six papers related to dental preclinical psychomotor skills in an educational setting, none of which were deemed high-quality. Deficiencies in these papers included no statements defining underpinning educational theory, limited acknowledgement of evidence-based simulation activities including preparation, briefing, simulation, feedback, debriefing, reflection and evaluation. Given the widespread use of simulation in dentistry, academics should be encouraged to publish their scholarly activities in simulation-based dental education in order so that all dental faculties can work towards developing contemporary simulation curriculum to provide optimum teaching and learning opportunities for students.

## Introduction

There is extensive evidence to support the use of simulation-based education across various disciplines of healthcare education ( Gaba, 2004). Structured activities or simulated-based learning experiences are implemented to develop new skills, manage specialised scenarios, introduce unfamiliar environments, and assess skills and attributes ( Reese, Jeffries and Engum, 2010; Health Education Training Institute, 2014; Williams and Song, 2016; Australian Commission on Safety and Quality in Health Care, September 2012). Significant advancement in simulation has been evident since the introduction of the patient safety movement and the primary focus on patient-centred care ( Gaba, 2004; Weller *et al.*, 2012; Williams and Song, 2016).

Within simulation-based education there are numerous modalities to allow for structured scenarios that encourage the development of knowledge synthesis and skill development. Modalities include simulated-based learning experiences, simulation activities, written scenarios, mannequins, role-play, immersive simulation and the use of anatomical feature representations known as part-task trainers ( Lopreiato *et al.*, 2016). There is a plethora of published and grey literature about healthcare simulation, with most papers associated with medicine and nursing ( Laschinger *et al.*, 2008).

Over the last five years, healthcare simulation-based education has adopted ‘deliberate practice’ for the purpose of education, training, general and competency-based assessment ( Gaba, 2004; Levine *et al.*, 2014). This evidence-based simulation learning is grounded in educational theory with the program design including a cycle of phases, where each phase is dependent on each of the other phases ( Seropian *et al.*, 2004). The phases include preparation, briefing (pre-briefing), the simulation activity, feedback, debriefing, evaluation and reflection. The learner is required to prepare for the simulation session by acquiring underpinning knowledge in order to participate in the simulation-based learning experience. The preparation is followed by a briefing (pre-briefing) of important information including the learning outcomes for the session. After the briefing (pre-briefing) session the learners progress to the simulation-learning activity, followed by feedback, debriefing and reflection. The entire cycle is evaluated to assess for learner reaction and satisfaction, acquisition of skills, learner behaviour, actions and post-simulation results.

A simulation program design based on educational theories makes simulation an ideal modality for training, professional development and formative, summative and high-stakes assessments. Simulation based education is a valid teaching and learning modality for students and practitioners in healthcare delivery ( Health Education Training Institute, 2014). All phases should be included to complete the learning loop ( Levett-Jones and Lapkin, 2014). The learners and simulation educators are involved in the phases from the initial simulation scenario preparation to the debrief and reflection at the completion of the loop. All phases of the simulation activity play a significant role, with some research suggesting that the debrief phase is more beneficial than the actual simulation procedural activity ( Levett-Jones and Lapkin, 2014).

It is well documented that dental and oral health education has utilised simulation for training for some time (HealthWorkforce Australia, 2010; Perry, Bridges and Burrow, 2015). Dental simulation has been in existence since 1894, when Oswald Fergus designed the first dental simulator known as ‘the phantom head’ ( Perry, Bridges and Burrow, 2015). Dentistry and oral health students use simulation for preclinical skills acquisition including cavity preparations, restorations, root canal and pulp therapy, extractions, orthodontics, and preventive dentistry such as debridement of calculus, removal of dental plaque, pit and fissure sealants, and fluoride applications (HealthWorkforce Australia, 2010). Dentistry and oral health simulation has advanced from the phantom head to include more detailed and complex mannequins, part-task trainers including teeth models, virtual reality and force-feedback haptic devices ( Wierinck *et al.*, 2007; HealthWorkforce Australia, 2010; Urbankova and Engebretson, 2011b; Urbankova and Engebretson, 2011a; Ben-Gal *et al.*, 2013; Fugill, 2013; Urbankova, Eber and Engebretson, 2013).

It has been reported that despite the requirement for simulation content within dental and oral health curricula in Australia, dental and oral health simulation programs vary widely with respect to content, delivery and contact hours (HealthWorkforce Australia, 2010; Commonwealth Government, 2014; Australian Dental Council, 2017). The New South Wales Health Education and Training Institute (NSW HETI) also confirmed that disparities exist between teaching and learning organisations in relation to simulation-based education ( Health Education Training Institute, 2014). The degree to which dental and oral health simulation curricula has been designed according to current best practice guidelines incorporating the cycle of phases is unknown. The aim of this literature review is to identify and analyse the existing research relating to the educational structure of dental and oral health simulation activities.

## Methods

Searching the literature can be achieved using several valid methods. One method is to perform a scoping review. Scoping reviews determine the extent of the existing literature relating to a chosen topic ( Arksey and O’Malley, 2007; Williams and Song, 2016). Scoping review methodology is appropriate when a body of literature has not been comprehensively reviewed or is of a diverse nature ( Khalil *et al.*, 2016). Although with a similar purpose to a systematic review methodology, a scoping study captures a broader range of studies with greater variation in study designs. The scoping review methodology is appropriate when the search results in a variety of different studies with potential inclusion criteria, and allows for the research question to be less specific, giving leniency for a broader range of literature to be included ( Arksey and O’Malley, 2007).

The scoping review was undertaken to identify the published literature and the grey literature relating to simulation-based dental education ( Levac, Colquhoun and O’Brien, 2010). The modified five stage methodological framework was followed to undertake this scoping review; this evidence-based method is outlined by Khalil and colleagues ( Khalil *et al.*, 2016) and based on previous studies by Arksey and O’Malley ( Arksey and O’Malley, 2007) and Levac and colleagues ( Levac, Colquhoun and O’Brien, 2010). The quality of the articles was assessed against the standards outlined by Schaefer and colleagues ( Schaefer *et al.*, 2011) the Consolidated Standard of Reporting Trials (CONSORT) and Strengthening the Reporting of Observational Studies in Epidemiology (STROBE) Statements ( Cheng *et al.*, 2016).

### Identification of the research question

This scoping review was conducted to answer the following research question: What educational theories and designs are implemented to teach dental and oral health preclinical psychomotor skills in the simulated learning environment?

### Identification of relevant studies

Medical subject headings (MeSH) were selected and confirmed based on the Joanna Briggs Institute (JBI) three-step method to search for studies ( Aromataris and Riitano, 2014). Firstly, a rudimentary search of the medical electronic databases MEDLINE and CINAHL was conducted using text words contained in the title, abstract and body of the articles. Upon completion of the initial search keywords were established and relevant studies were searched for using the University of Newcastle (UON) library and interlibrary services. The medical search engine electronic databases were accessed via the UON library website and included EMBASE, Scopus, PubMed including literature from MEDLINE, CINAHL complete and Web of Science. The final search stage included reading the reference lists of each retrieved study.

The keywords used were simulation, oral health, dental, students, healthcare and psychomotor. Truncation and Boolean operators were used to capture variations of search terms as shown in Tables 1 and 2. Inclusion criteria were set to include articles from all countries, published in English language, between 2005 and 2016. The inclusion dates were set to coincide with the introduction of oral health degrees in Australia.

**Table 1.** Keywords and Truncation

Search words used	Truncation options
simulat* ‘oral health’ dent* student* psychomotor ‘preclinical skill*’	simulation, simulating, simulate, simulated dental, dentist, dentistry students preclinical skills

**Table 2.** Search Strategies and Boolean operators

Number	Searches
1.	simulat*
2.	‘oral health’
3.	dent*
4.	2 or 3
5.	student*
6.	psychomotor
7.	‘preclinical skill*’
8.	6 or 7
9.	1 and 4 and 5 and 8

### Study selection

The study selection inclusion criteria were: the study population involved dental or oral health students, participating in preliminary preclinical simulated learning education, dental or oral health degree curriculum, teaching and learning preclinical simulation skills or dental or oral health simulation.

The study selection exclusion criteria were: healthcare students other than dental or oral health students, non-students and practising clinicians, advanced students honing preclinical simulated learning skills, dental specialty curriculum including surgical skills, pre and post assessments without an education component and healthcare simulation other than dental or oral health.

A study was included if it met the inclusion criteria and did not include the exclusion criteria.

### Charting the data

**Figure 1.**
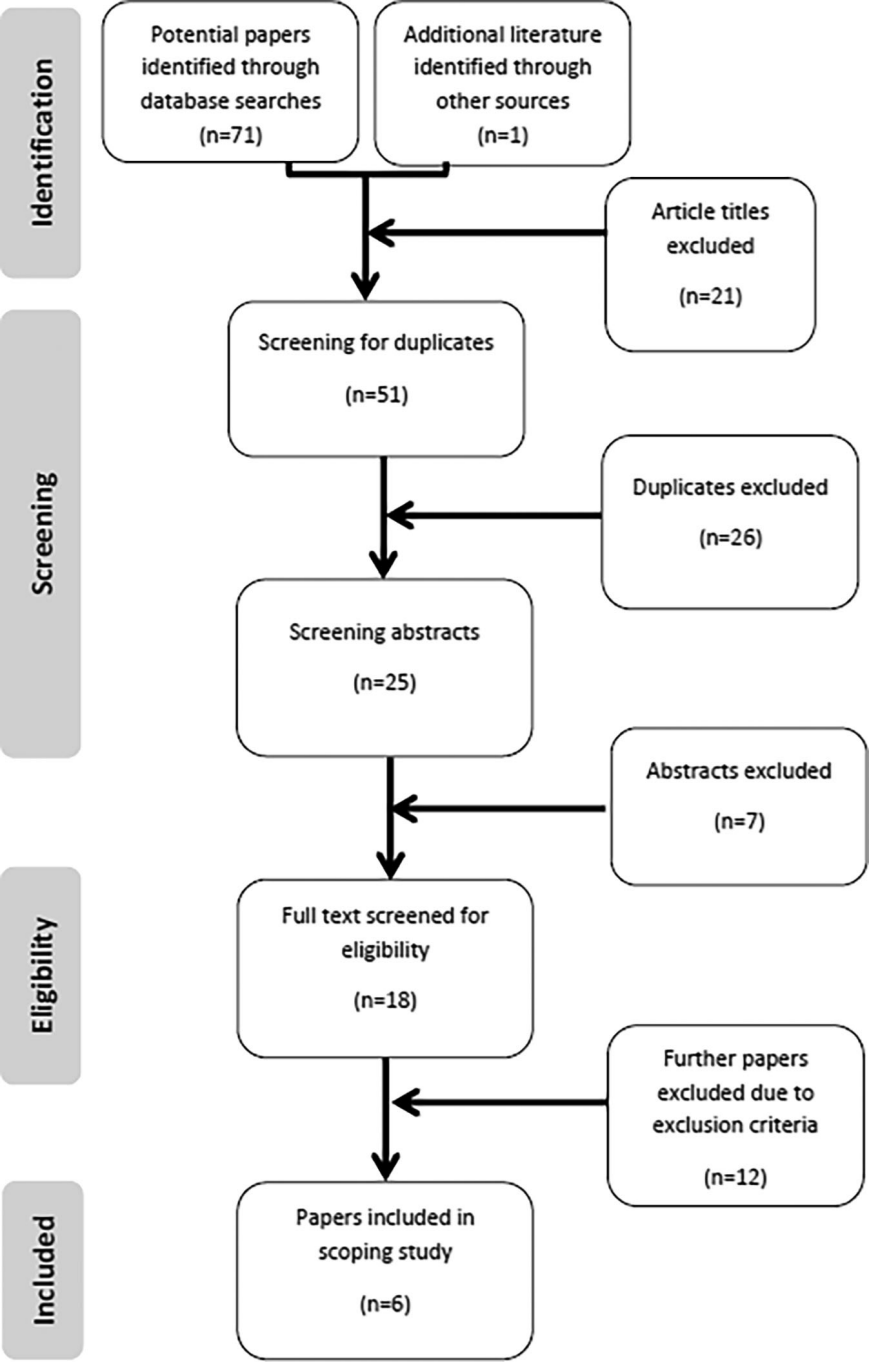
PRISMA flowchart

Figure 1 illustrates the process of identifying potential papers and screening them for inclusion of exclusion in the scoping review, using the Prisma flowchart. The details of the final six included papers are listed in Table 3.

## Results

The literature searching phase identified 72 potential papers. Article titles were read and 21 papers were eliminated due to the title not containing words relating to the inclusion criteria. The remaining 51 articles were screened resulting in the omission of 26 duplicates and seven irrelevant abstracts. A total of 18 full text papers were read to satisfy the eligibility process. Inclusion and exclusion criteria were applied reducing the total by a further 12 articles. Three articles were rejected because the studies involved either pre-test or post-tests to determine a correlation with preclinical operative dentistry examination scores and did not include discussion of an education component ( Urbankova and Engebretson, 2011b; Urbankova and Engebretson, 2011a; Urbankova, Eber and Engebretson, 2013). One paper was eliminated as 14 percent of the participants “were not dentists” and involved 12 test tasks ( Ben-Gal *et al.*, 2013). Another was excluded because the study involved final year dental students participating in a systematic carving exercise. The study was classified as honing pre-existing ability as opposed to acquiring new skills ( Kilistoff *et al.*, 2013). The final process resulted in a total of six included articles ( Welk *et al.*, 2008; Amer *et al.*, 2011; Gottlieb *et al.*, 2011; Fugill, 2013; Arigbede, Denloye and Dosumu, 2015; Walker and von Bergmann, 2015). Table 3 presents the first author’s name and year of paper, study population, study aims and design, simulation activity, outcomes measured and a summary of the results for all included articles.

**Table 3.** Articles selected for inclusion

Firstauthor	Study pop	Study aims	Study design	Activity	Outcomes measured	Results
Fugill 2013	Nil	Review article	Review	Preclinical skills	Simulation control and standardisation. Simulation simplification through handpiece manipulation and procedural skills. Controlled environment resulting in increased patient safety and developing skills at a competent and confident level.	Sim is a vital part of learning restorative dentistry. Sim contributes to patient safety. Dental education will need to replace patient care with more sim. Dental education using the apprenticeship model in most countries. There is an urgent need to understand the possibilities and limitations of sim
Walker 2015	2nd year dental students	Understand whether cognitive task analysis can help dental educators systematically identify steps & critical incidents in performing a preclinical dental task.	Pilot study	Class II wax carving of 25. Video of learning the procedure and post-simulation interview.	Understand the areas of the task that the students struggled with. Assess if there is any overlap of critical incidents between students and experienced clinicians.	CTA is useful in systematically identifying cognitively challenging incidents in performing a Class II wax carving in a sim environment
Amer 2011	1st year dental students	Compare performance on both written & practical skill evaluation of 1st year dental students receiving video instruction versus IDVG skill sets.	RCT	1st year dental students receiving conventional passive clinical video instruction versus interactive learning module teaching	Compare the clinical knowledge and performance of 1st year dental students receiving 2 differing educational approaches.	Results using an IDVG are as good as passive non-interactive teaching. Dental students prefer learning from IDVG as opposed to a lecture
Gottlieb 2011	Preclinical Faculty members	Explore Faculty members’ perceptions & expectations of students’ abilities in preclinical operative dentistry with and without VR simulation training	Survey	Non-VRS simulation laboratory for 1st year dental students for continued instruction in operative preparations and restorations	Preclinical Faculty members’ perception on non-VRS abilities at the end of the preclinical non-VRS course after having completed the preclinical VRS course.	VRS trained students may have an advantage in the clinical setting compared to non-VRS trained students
Welk 2008	1st year dental students	Discusses approaches used by University of Tennessee, College of Dentistry Health Science Center in using 40 DentSim units	Evaluation data	Maxillary and mandibular cavity preparations using DentSim and conventional simulation units	Data collected for computer technology knowledge, experience, incidental learning rate and DentSim introduction. Evaluation of elective training time, wearing headphones, acquisition and transfer effect of cognitive / motor skills	CAL and sim is a promising dental education method. Integrating 40 units is a challenge for Faculty and students. A step-by-step approach is required
Arigbede 2015	Final year dental students	Determine dental operative clinical skills teaching & learning practices in Southern Nigeria dental schools’ clinical skills labs & identify challenges to effective learning experience in clinical skills labs	Cross-sectional study	Routine final year clinical skills simulation learning environment session	Quantitative participant data for existing teaching and learning practices in dental skills laboratory and the transferability of skills to real clinical practice	Teaching and learning conforms to standard practice. Inappropriate feedback. Video demonstration non-existent. Equipment breakdown. Clinical instructors require continuous training in biomedical education

## Discussion

As previously mentioned, the quality of the articles was assessed against the standards of Schaefer and colleagues ( Schaefer *et al.*, 2011), and the reporting guidelines for CONSORT and STROBE ( Cheng *et al.*, 2016). Cheng and colleagues provided three detailed tables that outlined the key elements and extension elements to report for randomised control trails, observational trials and simulation-based research ( Cheng *et al.*, 2016). None of the six studies complied with the inclusion requirements for simulation-based research reporting.

Four of the included studies were conducted in Iowa ( Amer *et al.*, 2011), Pennsylvania ( Gottlieb *et al.*, 2011), Tennessee ( Welk *et al.*, 2008) and Southern Nigeria ( Arigbede, Denloye and Dosumu, 2015). The remaining two either did not state the location ( Walker and von Bergmann, 2015) or it was not applicable ( Fugill, 2013). Of the six included studies, no two were similar. All studies varied in aims, simulated clinical experience and simulation modalities. The study variations render it difficult to compare outcomes, although common themes were able to be extracted across the studies. Perception, preclinical skills, simulation modalities, educational theory, simulation phases and learners were identified as the common topics discussed in the six included studies.

### Simulation modalities

Simulation modalities vary in dental and oral health simulation. Fugill (2013) referred to the original simulation modality from 1894 known as the ‘phantom head’. The phantom head is a mounted part-task trainer complete with articulating jaws to practice preclinical dental and oral health skills ( Perry, Bridges and Burrow, 2015). Dental and oral health simulator development has led to the utilisation of technology including virtual reality (VR), computer assisted learning (CAL) and computer assisted simulation (CAS). Amer and colleagues (2011) compared the results of theoretical and practical skill assessment of first year dental students who participated in the Interactive Dental Video Game (IDVG) versus video. Gottlieb and colleagues (2011) analysed Faculty members’ perception and expectations of students’ ability after using Virtual Reality Simulation (VRS). Walker and colleagues (2015) utilised video to conduct cognitive task analysis.

Arigbede and colleagues (2015), and Welk and colleagues (2008) conducted studies that were designed to be carried out using the existing curriculum in the simulation laboratory. The aim of the study conducted by Arigbede and colleagues was to determine the current teaching and learning practices for preclinical dentistry. The details of the simulation modality were not identified, however the participants response highlighted equipment breakdown regularly ( Arigbede, Denloye and Dosumu, 2015). Dental equipment in a simulation laboratory would usually include a dental mannequin and part-task trainer simulators. Welk and colleagues described and discussed the approach on the use of 40 computer assisted dental patient simulators known as DentSim ( Welk *et al.*, 2008).

Professional areas under assessment based on opinion

Fugill’s (2013) review paper did not involve participants, whereby the article was a review article that gathered information pertaining to standardised simulation delivery and a review of ‘procedural skills’ as a general topic with no direct specification to a psychomotor skill or the teaching and learning methodology. The Gottlieb and colleagues (2011) study involved the participants, the preclinical Faculty members, providing responses in a survey based on their perceptions. The participants of the other four studies were dental students ranging from first year through to final year ( Welk *et al.*, 2008; Amer *et al.*, 2011; Arigbede, Denloye and Dosumu, 2015; Walker and von Bergmann, 2015).

### Perception

Non-measurable outcomes such as confidence, perception, expectation, ideas and experience were reported in three studies. Fugill (2013) surmised that simulated learning increases student confidence. Two studies reported on perception, expectation, ideas and experience ( Gottlieb *et al.*, 2011; Arigbede, Denloye and Dosumu, 2015); Dental faculty members reported their perceptions and expectations of students’ preclinical dental operative ability in the study by Gottlieb *et al.* (2011) However, the study did not include quantitative data on measurable student outcomes. The faculty expected an increase in student performance ability and confidence in the simulation laboratory from the students who participated in the virtual reality simulation (VRS) modality. The same study noted that Faculty anticipated the VRS participants would demonstrate a higher stress level. ( Gottlieb *et al.*, 2011)

Virtual reality simulation was perceived to generally increase student preparation and self-assessment abilities ( Gottlieb *et al.*, 2011). Knowing and developing faculty perception of VRS has been identified as critical in the design of preclinical dental simulation curriculum ( Gottlieb *et al.*, 2011). Students preferred learning and acquiring skills with the aid of mixed modalities including computer assisted learning (CAL) and interactive dental video games (IDVG) ( Welk *et al.*, 2008; Amer *et al.*, 2011); further, it has been reported elsewhere that the use of technology can encourage constructive learning ( Shah and Cunningham, 2009).

Welk and colleagues (2008) reported a reduction in the compulsory course time and increased free time for students to access the simulation laboratory. The paper implied, but did not confirm, that increased efficiency may be an outcome as a result of CAL. It is worth noting that the study did not provide methodology for the simulation laboratory free time.

### Preclinical skills

The preclinical skills simulation task underpinning each of the six studies varied. Four of the six studies utilised dental students as participants ( Welk *et al.*, 2008; Amer *et al.*, 2011; Arigbede, Denloye and Dosumu, 2015; Walker and von Bergmann, 2015) and the curriculum from first, ( Welk *et al.*, 2008; Amer *et al.*, 2011) second ( Walker and von Bergmann, 2015) and ‘final’ year courses. Preclinical operative dentistry including resin bonding ( Amer *et al.*, 2011), dental cavity preparations ( Welk *et al.*, 2008; Gottlieb *et al.*, 2011) and carving dental restorations ( Walker and von Bergmann, 2015) were the selected simulation tasks. The two remaining studies reviewed and analysed the dental curriculum generally and did not report on a specific task ( Fugill, 2013; Arigbede, Denloye and Dosumu, 2015).

All the authors recommended integrating simulation into existing dental curriculum to enhance the teaching and learning effect. The studies recommended simulation tasks such as psychomotor skills ( Gottlieb *et al.*, 2011; Walker and von Bergmann, 2015), task repetition, cognitive task analysis ( Walker and von Bergmann, 2015), faculty perception of ergonomics, finger rests, student positioning and use of hand instruments including dental mouth mirror ( Gottlieb *et al.*, 2011). The authors recommend to integrate technology ( Welk *et al.*, 2008; Amer *et al.*, 2011), evaluate faculty positions to increase expert educators, provide instructional videos ( Arigbede, Denloye and Dosumu, 2015), include dyad learning at a novice and beginner learner level ( Walker and von Bergmann, 2015), increase knowledge and awareness of dental and oral health simulation ( Fugill, 2013), and confirm clinical performance in the simulation environment ( Gottlieb *et al.*, 2011). The studies unanimously suggested that further research is required in dental and oral health simulation.

### Educational theory

Educational pedagogy is fundamental to teaching design in higher education. Healthcare simulation program designs require sound educational theory, effective simulation practice and evidence-based discipline content ( Schaefer *et al.*, 2011; Weller *et al.*, 2012). A healthcare simulation literature review by Schaefer and colleagues (2011) looked at educational theoretical frameworks in addition to other criteria. The review was unable to draw any inferences or conclusions due to poorly designed studies. The same was found during the literature search for this scoping review. The six included studies did not state the educational theory that framed their study. Walker and Bergmann (2015) described the participants practising psychomotor skills associated with a Class II wax carving restoration eight to nine times prior to the research, however the study did not specify when the practice took place. Amer and colleagues (2011) alluded in the methodology section that the participants moved through the stages when the task was performed adequately but provided no standard. Gottlieb and colleagues (2011) stated hours of course time completed, however did not link the time with the study. Welk and colleagues (2008) referred to the simulation task as a psychomotor skill but did not elaborate to describe the underpinning educational theory. Four out of six studies implied the educational theory of deliberate practice would be the most appropriate framework ( Welk *et al.*, 2008; Amer *et al.*, 2011; Gottlieb *et al.*, 2011; Walker and von Bergmann, 2015).

### Simulation phases

Evidence-based healthcare simulation includes sequential phases to prepare the learner for the simulation task ( Seropian *et al.*, 2004). The model continues with feedback and debriefing to complete the learning loop ( Seropian *et al.*, 2004). The study by Arigbede and colleagues (2015) documented preparation in the form of in-class lectures. The simulation phase continued with a demonstration of the task, step-by-step tutor instructions and feedback during and after the simulation task. The study mentioned self-assessment and reflection as an intermittent stage that may occur prior to grading the task. The study concluded that the delivery of video demonstration is non-existent and outlined that knowledge and skills can be supported with instructional videos. ( Arigbede, Denloye and Dosumu, 2015) Amer and colleagues (2011) noted learner preparation in the form of a didactic lecture. The IDVG modality was developed to read the task instructions prior to proceeding to the next stage, however, the study did not mention the healthcare simulation stages of briefing, feedback, debriefing, reflection or the use of a valid tool for simulation program evaluation. Walker and von Burgmann (2015) outlined the initial phase of the simulation without referring to it as preparation. Faculty were consulted to determine the ideal task to analyse. The student participants had learned the task in advance and were given the opportunity to practice the task at least nine times. The study did not mention the briefing phase, and debriefing was referred to as ‘an interview’ of 90 minutes in duration. Feedback was provided from dental expert one (DE1) to dental expert two (DE2) and in video format for student benefit. The study by Gottlieb and colleagues (2011) was based on Faculty perception and expectation of the students. The study data is not based on first hand clinical ability. The study included information on preparation in the methods section, however did not mention briefing, debriefing and reflective phases. Welk and colleagues (2008) comprehensively included preparation in the form of lectures, feedback given by the CAL DentSim software and step-by-step checklist for guided practice. Specific mention of a briefing was not made, however learning outcomes were identified and extension preparation was delivered prior to the DentSim simulated learning exercise. The study did not mention self-reflection or evaluation of the program design.

### Dental and oral health learners

All of the six included studies involved dental schools. The participants were dental students ( Welk *et al.*, 2008; Amer *et al.*, 2011; Arigbede, Denloye and Dosumu, 2015; Walker and von Bergmann, 2015) and dental faculty members ( Gottlieb *et al.*, 2011). The review by Fugill (2013) comprehensively reviewed dental simulation including standardisation, patient safety and transfer of clinical skills. The review did not involve participants or a location. ( Fugill, 2013)

Future research is suggested in the forms of evaluating the potential of simulation technology, ( Amer *et al.*, 2011) clinical performance and attitude to new technology ( Gottlieb *et al.*, 2011) and possibilities and limitations of simulation ( Fugill, 2013). Fugill (2013) confidently proposed the prospect of simulation replacing a portion of patient care education. Other healthcare simulation programs have successfully shown that simulation can replace up to 25 percent of patient care model of education ( Watson *et al.*, 2012).

### Limitations and future research

There were no studies retrieved or included with oral health student participants ( Welk *et al.*, 2008; Amer *et al.*, 2011; Gottlieb *et al.*, 2011; Fugill, 2013; Arigbede, Denloye and Dosumu, 2015; Walker and von Bergmann, 2015). The limitations of this scoping review include the wide variation of the studies. The inclusion criteria included papers published from 2005 and 2016. When the reference lists were hand searched there were numerous articles predating 2005, however, earlier articles were not included due to the advancement of simulation in healthcare over the last 30 years ( Gaba, 2004) and the lack of articles pertaining to dental and oral health.

Some of the studies included reference to one or a combination of the phases of simulation ( Welk *et al.*, 2008; Amer *et al.*, 2011; Gottlieb *et al.*, 2011; Arigbede, Denloye and Dosumu, 2015; Walker and von Bergmann, 2015), however none included detail of all phases of simulation. Reference to simulation as a technique was not mentioned in the studies. Often the word ‘simulation’ was used in varying contexts throughout the included articles and was not defined as either the ‘simulation technique’ or as ‘simulator technology’.

Future research should look at dental and oral health simulation-based education programs designed with educational theory. Research should also focus on dental and oral health simulation-based education program evaluation to meet education, assessment and safety standards (Australian Commission on Safety and Quality in Health Care, September 2012) and address documented gaps between current practice and best outcomes.

## Conclusion

This scoping review has addressed the research question “What educational theories and designs are implemented to teach dental and oral health preclinical psychomotor skills in the simulated learning environment?” and found little evidence of any educational theories and designs being implemented in the design of simulation curricula in dentistry and oral health. Simulation program designs should be developed using educational theory, evidence-based content and evidence-based simulation ( Schaefer *et al.*, 2011). There is little data to support that educational theory is embedded in dental and oral health preclinical skills simulation curriculum. The published literature appears vague about the details of clinical and non-clinical simulation curriculum. Future research should look the design of simulation-based dental education programs with educational theory, and address documented gaps between current practice and best outcomes.

## Take Home Messages

• There is little evidence of educational theories and designs being implemented in the design of simulation curricula in dental education.
• Deficiencies in the existing research included no statements defining underpinning educational theory, limited acknowledgement of evidence-based simulation activities including preparation, briefing, simulation, feedback, debriefing, reflection and evaluation.
• The outcomes of this literature review have located dental and oral health simulation curriculum design relative to current best-practice guidelines used in other healthcare disciplines.
• The ability to highlight any deficiencies in the structure of existing simulation-based education in dental programs will facilitate improved design of the simulation curriculum in dental education.

## Notes On Contributors

Dr Denise Higgins is a Lecturer and Program Convenor (Oral Health) at the University of Newcastle. Denise is an innovator of fit-for-purpose simulation technology to enhance the student experience in the educational environment. Denise’s creations include the design and development of a synthetic tissue simulator capable of repeated needle insertion and dental local anaesthetic deposition.

Dr Melanie Hayes is a Senior Lecturer in the Work Integrated Learning team in the School of Health Sciences, Faculty of Medicine and Health at The University of Sydney. Her scholarship focuses on health professional education, including preparedness for practice and career development. ORCID: https://orcid.org/0000-0003-1001-1267

Professor Jane Taylor is a Conjoint Professor of Oral Health at the University of Newcastle. She is a qualified dentist with specialist training in Forensic Odontology. As previous Discipline Lead in Oral Health she spearheaded the expanding research activities of this emerging discipline.

Associate Professor Janet Wallace is the Head of Discipline (Oral Health) at the University of Newcastle. Her research expertise includes evaluating student placements, service -learning evaluation, and oral health therapy teaching. She is the Chief Investigator and Architect of Senior Smiles, a preventive oral health care program for older people living in residential aged care facilities.

## Declarations

The author has declared that there are no conflicts of interest.

## Ethics Statement

Not required as it is a review of the literature.

## External Funding

This article has not had any External Funding
