# Supplementary material for: A scoping review of simulation-based dental education
Source: MedEdPublish (2016). 2020 Feb 27;9:36. [Version 1] doi: 10.15694/mep.2020.000036.1 (PMC10697570; doi:10.15694/mep.2020.000036.1)
Supplement: Peer review history

# Reviewer report: Reviewer response for version 1

**DOI:** 10.21956/mep.20010.r30508

**Published:** 2020-06-28

**Authors:** Megan Anakin

**Affiliations:** 1 University of Otago

This review has been migrated. The reviewer awarded 3 stars out of 5 Thank you for publishing this scoping review about simulation-based dental education. In the introduction, the authors cite many sources to explain that simulation is well-established in healthcare education. Further elaboration in several areas would help the reader to better appreciation the warrant for this study. For example, the authors state that “There is a plethora of published and grey literature…”, however, perhaps the authors might consider stating what this literature tell us about healthcare simulation that is relevant to the need for this scoping review. Another example is, “This evidence-based simulation learning is grounded in educational theory…” and “ A simulation program design based on educational theories…”, where if the authors specify the educational theory or theories then it will help them to develop their argument for the study and its relevance to dental and oral health education will be strengthened. A third example, in the fifth paragraph of the introduction is a statement about ‘disparities’ in simulation-based education. Please consider describing these disparities so the reader can understand the issues that this scoping review hopes to address. The authors may wish to describe the main elements of the ‘current best practice guidelines’ since they state that they are the reference point for comparing the findings of their literature review. To enhance understanding of the methodological framework used to perform the scoping review, the authors may wish to describe how they carried out the five steps with the standards. The authors may wish to consider outlining the educational theories and designs used in other areas of healthcare simulation in the introduction so this information can used in the methods section to describe how it was incorporated into the methodological framework. The search strategy is well-described, however, the bulk of the results contains a description of how the final set of articles were chosen and this information belongs in the methods section. In the results section, the six selected article are presented in Table 3, however, the results of the analysis that addresses the research question, “What educational theories and designs are implemented to teach dental and oral health preclinical psychomotor skills in the simulated learning environment?” and the best practice guidelines are not presented in the results section. The information in the first two paragraphs of the discussion report demographic information about the six articles, and the seven subsections present the results of the analysis, therefore, the authors may wish to move this information into the results section. This move would allow space in the discussion for the authors to summarise their main findings and discuss the significance of them in relation to the literature presented in the introduction. The authors may wish to read from Lingard, L. (2015). The art of limitations. Perspectives on Medical Education, 4(3), 136-137 to enhance their discussion of the limitations of their study. I encourage the authors to consider submitting a revised version of this article so readers can better appreciate their study and its findings.

Reviewer Expertise:

NA

No decision status is available

# Reviewer report: Reviewer response for version 1

**DOI:** 10.21956/mep.20010.r30507

**Published:** 2020-05-19

**Authors:** Ken Masters

**Affiliations:** 1 Sultan Qaboos University

This review has been migrated. The reviewer awarded 3 stars out of 5 An interesting scoping review of simulation-based dental education. The paper is mostly very well written – beginning with an Introduction that clearly details the value of simulation training in medical education in general, and then going into the detailed description of the Methods. There are issues that need to be addressed, however, mostly related to the structure of the paper:• The Research Question is placed in the Methods. I think it would be better to be placed at the end of the Introduction (or at the very beginning of the Methods), with the logic that the Methods are the processes followed to answer the research question.• The order of the papers in Table 3 appears to be random. They really should be in alphabetical order of first author surname (or, perhaps, year). If the authors are using a different order, then this should be stated and supporting reason(s) given.• Given that the focus of the paper was on the extent of theory in the studies, I would have expected Table 3 (or a new table) to describe the extent to which theory is employed. (I understand that it was minimal, and this is described in the Discussion (under the sub-heading “Educational Theory”), but they really should be first presented in the Results.) The Discussion should then discuss these results in light of the literature.• Elsewhere also, I see several results first presented in the Discussion, so perhaps it would be a good idea for the authors to go through the entire Discussion, ensuring that all results are first presented in the Results section, and then discussed in the Discussion.• In Figure 1, the arrows to the exclusion boxes should be pointing in the opposite direction.So, overall, there is a great deal of useful information in the paper, but there is some re-structuring required in order to make that information more accessible to the reader.

Reviewer Expertise:

NA

No decision status is available

# Reviewer report: Reviewer response for version 1

**DOI:** 10.21956/mep.20010.r30509

**Published:** 2020-04-15

**Authors:** Trevor Gibbs

**Affiliations:** 1 AMEE

This review has been migrated. The reviewer awarded 4 stars out of 5 An interesting and well written paper, that opens up a field of opportunity for further research. As someone who travels extensively and observes many curricula and teaching methodologies, it is easy to get a biased view of a subject such as dental simulation and be impressed with the new technologies that are evolving in dental simulation. It is interesting therefore to read that much of the innovation lacks the theoretical basis that it probably deserves and needs.I hope that this piece of research opens up much more discussion on the topic and promotes a grounded basis for dental simulation. I would recommend this paper to all those involved with developing dental curricula.

Reviewer Expertise:

NA

No decision status is available

# Reviewer report: Reviewer response for version 1

**DOI:** 10.21956/mep.20010.r30510

**Published:** 2020-03-07

**Authors:** Puja Dulloo

**Affiliations:** 1 Pramukhswami Medical College

This review has been migrated. The reviewer awarded 4 stars out of 5 The topic chosen by the author is related to the learning of psychomotor skills from the initial year (pre-clinical) of dental education. The abstract description is concise reflecting what has been done to present the conclusion accurately. The background of the study is well explained, highlighting the need for this review. It would have been better if the need for this study would have been mentioned clearly. The use of the Scoping review methodology for the study makes it different, where the focus was to identify and analyze the existing literature relating to the educational theories used to structure the simulation activities in dental and oral health, as per the research question framed for the study. This allows the author to identify the gap in the research and provide an opportunity for researchers to identify the key concepts for the topic. The methodology was well described as per the search engines' used for the literature search, although the Boolean operation used for searching strategies were not mentioned in table-2. In my opinion, the initial search should have reflected the number of articles searched in each search engine and then remove the number of duplications. Variability in the objectives, methodologies used, and responders included in the research studies chosen limited the authors to come up to a clear-cut conclusion. The review study focuses on the gap in the present literature available and provides a clear take-home message for the researches to move ahead with researches based on the use of the simulation technology and specific technique, highlighting the educational theory used for the simulation-based curriculum at pre-clinical level of dental students, with a focus on oral hygiene activities.

Reviewer Expertise:

NA

No decision status is available
